# Assessing the Health and Economic Impact of a Potential Menthol Cigarette Ban in New York City: a Modeling Study

**DOI:** 10.1007/s11524-021-00581-8

**Published:** 2021-11-09

**Authors:** Yan Li, Julia Sisti, Karen R. Flórez, Sandra S. Albrecht, Anita Viswanath, Marivel Davila, Jennifer Cantrell, Diksha Brahmbhatt, Azure B. Thompson, John Jasek, Earle C. Chambers

**Affiliations:** 1 Department of Population Health Science and Policy, Icahn School of Medicine at Mount Sinai, New York, NY 10029 USA; 2 NYC Department of Health and Mental Hygiene, New York, NY USA; 3 Environmental, Occupational, and Geospatial Sciences Department, CUNY School of Public Health and Health Policy, New York, NY USA; 4 Department of Epidemiology, Mailman School of Public Health at Columbia University, New York, NY USA; 5 Department of Epidemiology, NYU Global School of Public Health, New York, NY USA; 6 Department of Community Health Sciences, School of Public Health, SUNY Downstate Health Sciences University, Brooklyn, NY USA; 7 Department of Family and Social Medicine, Albert Einstein College of Medicine/Montefiore Medical Center, 1300 Morris Park Ave, Bronx, NY USA

**Keywords:** Tobacco control, Health disparity, Cardiovascular disease, Urban health

## Abstract

Menthol in cigarettes increases nicotine dependence and decreases the chances of successful smoking cessation. In New York City (NYC), nearly half of current smokers usually smoke menthol cigarettes. Female and non-Latino Black individuals were more likely to smoke menthol-flavored cigarettes compared to males and other races and ethnicities. Although the US Food and Drug Administration recently announced that it will ban menthol cigarettes, it is unclear how the policy would affect population health and health disparities in NYC. To inform potential policymaking, we used a microsimulation model of cardiovascular disease (CVD) to project the long-term health and economic impact of a potential menthol ban in NYC. Our model projected that there could be 57,232 (95% CI: 51,967–62,497) myocardial infarction (MI) cases and 52,195 (95% CI: 47,446–56,945) stroke cases per 1 million adult smokers in NYC over a 20-year period without the menthol ban policy. With the menthol ban policy, 2,862 MI cases and 1,983 stroke cases per 1 million adults could be averted over a 20-year period. The model also projected that an average of $1,836 in healthcare costs per person, or $1.62 billion among all adult smokers, could be saved over a 20-year period due to the implementation of a menthol ban policy. Results from subgroup analyses showed that women, particularly Black women, would have more reductions in adverse CVD outcomes from the potential implementation of the menthol ban policy compared to males and other racial and ethnic subgroups, which implies that the policy could reduce sex and racial and ethnic CVD disparities. Findings from our study provide policymakers with evidence to support policies that limit access to menthol cigarettes and potentially address racial and ethnic disparities in smoking-related disease burden.

## Introduction

Smoking is the leading preventable cause of premature death worldwide [1–3] and increases the risk for many adverse health outcomes including cancer, heart disease, stroke, and chronic obstructive pulmonary disease (COPD). [4–6] While overall smoking prevalence has decreased over the past two decades in the United States (US), there has been an increase in menthol cigarette sales and use among current smokers. [7–9] There is strong evidence that menthol in cigarettes increases nicotine dependence and decreases the chance of successful smoking cessation. [7, 10, 11]

Menthol cigarette use is particularly prevalent among females, youth, and people of color in the US, likely due in part to aggressive tobacco industry marketing and promotion to these communities. [7, 12–14] Women are more likely to smoke menthol-flavored cigarettes than men. [13] More than 50% of youth that smoke cigarettes smoke menthol-flavored cigarettes. [15] Almost one-third of current menthol smokers are Black compared to only 3% of non-menthol smokers who are Black. [16] Health advocacy agencies have come out strongly against the sale of flavored cigarettes because of their predominance of use among marginalized populations. [17, 18] The World Health Organization (WHO) recommends banning the use of menthol in cigarettes and other tobacco products in an effort to decrease the prevalence of smoking and improve population health. [19, 20] The US Food and Drug Administration (FDA) also recently announced that it will ban menthol—the last allowable flavor—in cigarettes. [21]

Beginning in January 2017, the province of Ontario, Canada, banned the use of menthol-flavored tobacco products. [22] Evaluation studies of this ban show a significant reduction in the sales of cigarettes in Ontario and an increased rate of quitting among daily and occasional menthol smokers. [22–24] New York City (NYC) has considered implementing a similar policy, building on its ban of non-menthol-flavored tobacco products; [25] legislation was introduced in 2019 but, in the face of opposition, was not put to a vote. [26] Although there is promising evidence of the effectiveness of a menthol cigarette ban, the long-term health and economic costs of these types of cigarette bans are unclear. It is also uncertain whether this ban will have similar effects among groups who experience tobacco-related health disparities, [27] specifically racial and ethnic minorities, a group targeted by the tobacco industry for the sale of menthol cigarettes and known to disproportionally use menthol cigarettes. [14]

To inform policymaking, this study aims to project the long-term health and economic impact of a potential menthol cigarette ban in NYC using a microsimulation model of cardiovascular disease (CVD). We use CVD as the outcome of interest, as it is the leading cause of death in the US and NYC, and smoking is one of the most important risk factors for CVD. [28] In addition, there are stark sex and racial and ethnic disparities in CVD risk factors in NYC and across the country. [29, 30] Since the prevalence of menthol cigarette use varies significantly across different sex and racial and ethnic groups, a second aim is to examine how the potential ban may differentially affect individuals across sex and race and ethnicity, including whether or not the policy would reduce disparities in health outcomes and healthcare costs between groups.

## Methods

We developed a microsimulation model of CVD that can be used to project the long-term impact of a public health policy or intervention on CVD health outcomes and healthcare costs. In a microsimulation model, simulated individuals are generated with predefined characteristics (e.g., age, sex, race, ethnicity). Their health behaviors (e.g., smoking, diet) can be modified to understand how such behaviors would impact health outcomes (e.g., myocardial infarction, stroke) as the simulation runs. [31, 32] By simulating the same group of individuals under different policy scenarios, researchers can compare the impact of different policies and identify the most cost-effective policy in a low-risk “virtual” environment.

### Model Structure

**Fig. 1:**
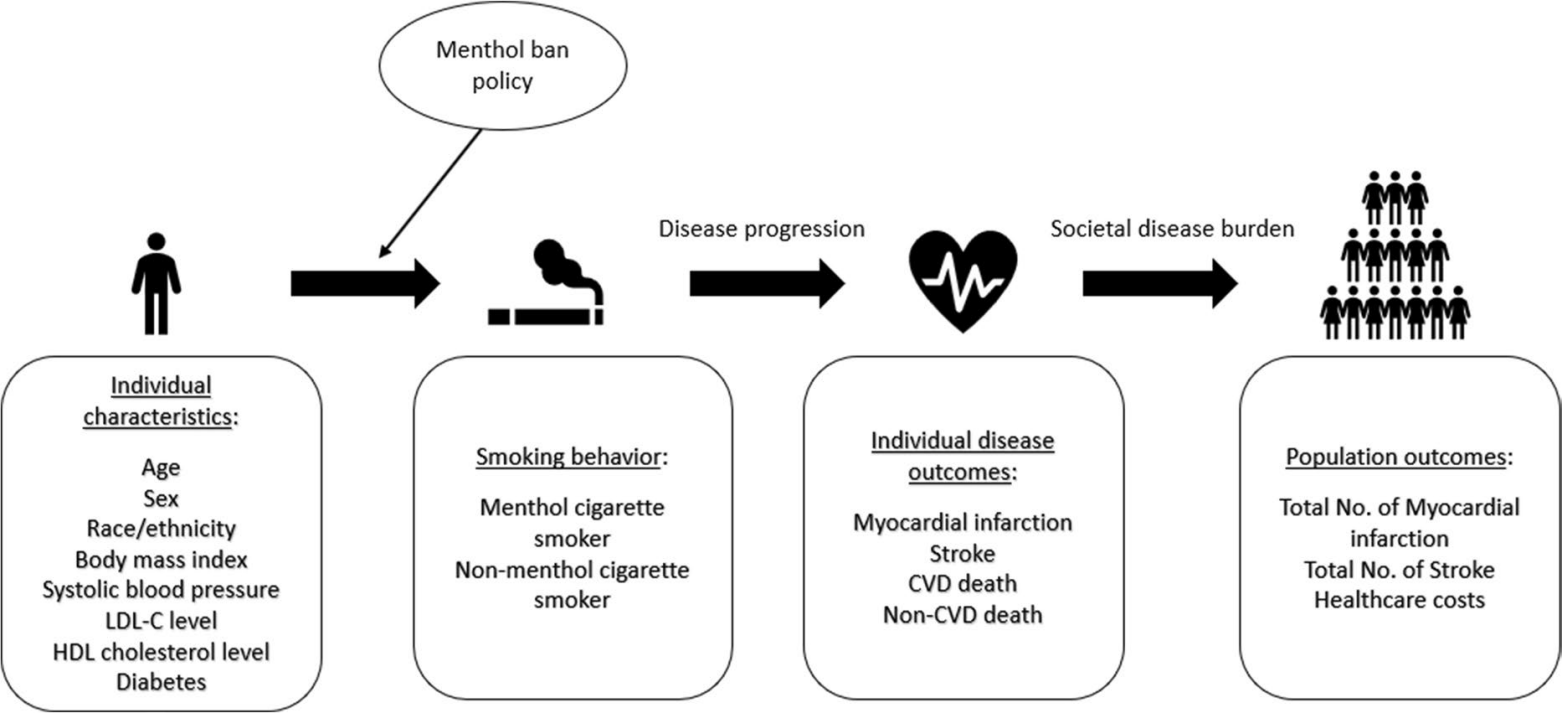
Model schematic. Notes: In the microsimulation model, NYC adult smokers with different demographic characteristics and health profiles will be generated first. Then each simulated individual will become either menthol cigarette smoker or non-menthol cigarette smoker based on a probability that is calculated based on his/her demographic characteristics. As the simulation runs, individuals will grow older and develop either myocardial infarction, stroke, or decease due to CVD or non-CVD reasons. Finally, the model will calculate the total numbers of myocardial infarction and stroke and cumulative healthcare costs due to CVD

The microsimulation model used for this study can project CVD outcomes (i.e., myocardial infarction (MI) and stroke) and healthcare costs among adults in NYC. Figure 1 shows the model schematic design. Specifically, the model can generate a group of adults with a variety of demographic characteristics. We only simulate adult cigarette smokers in this study because the menthol ban policy would primarily affect adults who smoke. Each simulated individual is assigned to be a menthol cigarette smoker or non-menthol cigarette smoker based on a probability that is calculated based on his/her sex and race and ethnicity. As the simulation runs, individuals will age and potentially develop CVD. Finally, the model will calculate the total numbers of MI and stroke and cumulative healthcare costs due to CVD. The model was programmed using simulation software AnyLogic 8. [33]

### Parameter Estimation

Publicly available health data are used from various city-based sources to inform our simulation models to estimate and calibrate the model parameters. Specifically, we estimated demographic and health characteristics based on data from the 2013–2014 NYC Health and Nutrition Examination Survey (HANES). The NYC HANES data, a cross-sectional population health survey designed to assess the health of New Yorkers, was used because it includes a range of variables on CVD and its risk factors. [34] It also provides a representative sample of non-institutionalized adults in NYC. Standardized protocols used in NHANES and testing laboratories were used in the NYC HANES to collect data for demographic and health factors.

The CVD risk factors included in the model are age, sex, race, ethnicity, body mass index, systolic blood pressure, LDL-C level, HDL cholesterol level, diabetes, and smoking status. Annual CVD event and CVD-related mortality probabilities were estimated using the Cox proportional hazard regression functions (Eq. 1) based on data from a National Heart, Lung, and Blood Institute Pooled Cohort. [35]

1 $$P\left(incident CVD event\right)= \frac{\mathrm{exp}\left(\alpha + {\beta }_{BMI}BMI+{\beta }_{LDL-c}{X}_{LDL-c}+\dots +{\beta }_{sbp}{X}_{sbp}\right)}{1+\mathrm{exp}\left(\alpha + {\beta }_{BMI}BMI+{\beta }_{LDL-c}{X}_{LDL-c}+\dots +{\beta }_{sbp}{X}_{sbp}\right)}$$

We estimated the prevalence of usual menthol cigarette use among current smokers using data from the 2018 NYC Community Health Survey, a cross-sectional, dual-frame cellphone/landline survey conducted annually by the NYC Department of Health and Mental Hygiene. [36] More specifically, responses to the following question “Thinking about the type of cigarettes you usually smoke, are they menthol or non-menthol?” were used to identify those who usually smoke menthol cigarettes in NYC. We then calculated the prevalence of usual menthol cigarette use across sex (i.e., male, female) and racial and ethnic (i.e., non-Latino White, non-Latino Black, Latino) groups.

If the menthol cigarette ban is to be implemented, the proportion of current menthol smokers who would quit was estimated to be 21.2% based on an evaluation of the menthol cigarette ban policy implemented in the province of Ontario, Canada. [23] The other menthol smokers (78.8%) may switch to non-menthol cigarettes or cigars or purchase menthol cigarettes in other cities where there is no such ban. [37] We used evidence from Ontario because it is the best available real-world evidence on the effect of a menthol cigarette ban, and uncertainty on the parameter estimation was assessed by sensitivity analyses. We also assumed that the menthol ban policy would only affect those who smoke menthol cigarettes, not those who smoke non-menthol cigarettes or did not smoke. This assumption was supported by findings from a recent expert elicitation on the effects of a menthol ban policy in the US. [38]

We estimated healthcare cost parameters based on the Medical Expenditure Panel Survey (MEPS) data. [39] Specifically, every simulated individual accrues annual age-specific “background” cost when he/she has not yet developed CVD. When the simulated individual develops MI or stroke, the specific cost associated with that disease will be added to the total healthcare costs. All healthcare costs were discounted at 3% (a widely accepted discount rate in economic evaluations of health policies) [40] and converted to 2018 dollars. Sensitivity analysis was conducted to assess the impact of different discount rates on healthcare costs.

### Simulation Experimental Design

Our study includes two simulation scenarios—implementing the menthol cigarette ban and not implementing the menthol cigarette ban (the status quo). For each of the scenarios, we simulated 10,000 adult smokers and projected the cumulative cases of MI and stroke as well as healthcare costs associated with CVD over a 20-year period (from 2018 to 2038). We calculated the averted cases of MI and stroke and healthcare cost savings among adult smokers if the menthol cigarette ban policy was implemented in NYC. We further calculated the reductions in the incidence of MI and stroke and associated healthcare costs across sex and racial and ethnic groups to examine the impact of the policy on health disparities in the long term.

## Results

Table 1 reports the prevalence of usually smoked menthol cigarettes among adult smokers in NYC in 2018. There were about 880,000 adult smokers, among which approximately 431,000 (49%) usually smoked menthol cigarettes. About 44.1% of male smokers usually smoked menthol cigarettes, while the percentage was 59.3% among female smokers (*p* = 0.001). One-quarter (25.6%) of non-Latino White adults usually smoked menthol cigarettes, compared with 83.8% among non-Latino Black adults and 59.8% among Latino adults (*p* < 0.001 for both groups). In addition, adults who usually smoke menthol cigarettes were more likely to have lower educational attainment and household income compared to those who usually smoke non-menthol cigarettes. There were no significant differences in the prevalence of usual menthol cigarette smoking across different age groups.

**Table 1:** Prevalence of usually smoked menthol cigarettes among NYC adults (18 +) smokers in 2018 (*N* = 880,000)

	**Weighted *n***	**%**	**95% CI**	***P*-value**
**NYC overall**	431,000	49.0	44.7–53.4	–
**Age**				
18–24	31,000	48.5	32.3–65.1	ref
25–44	194,000	50.3	43.7–56.9	0.850
45–64	181,000	55.0	48.7–61.2	0.487
65 and older	25,000	36.0	26.4–46.8	0.217
**Sex**				
Male	229,000	44.1	38.7–49.6	ref
Female	202,000	59.3	52.3–65.9	0.001
**Race/ethnicity**				
Non-Latino White	75,000	25.6	19.7–32.6	ref
Non-Latino Black	182,000	83.8	76.7–89.1	< .001
Latino	126,000	59.8	51.9–67.2	< .001
**Education level**				
Less than high school	102,000	51.2	41.3–60.9	< .001
High school graduate	138,000	60.5	52.2–68.2	< .001
Some college or technical school	134,000	53.5	45.6–61.2	< .001
College graduate or above	56,000	28.3	21.7–35.9	ref
**Household income**				
< 200%FPL	258,000	56.4	49.8–62.7	< .001
200 to < 400% FPL	111,000	52.5	44.1–60.7	< .001
400% + FPL	63,000	29.9	22.8–38.0	ref

Abbreviation: *FPL* federal poverty level

Data source: NYC Community Health Survey (CHS), 2018

(1) CHS 2018 data are weighted to the adult residential population per the American Community Survey, 2017. (2) Data are age-adjusted to the US 2000 Standard Population, except for age-specific estimates. (3) Population estimates are rounded to the nearest thousand

**Fig. 2:**
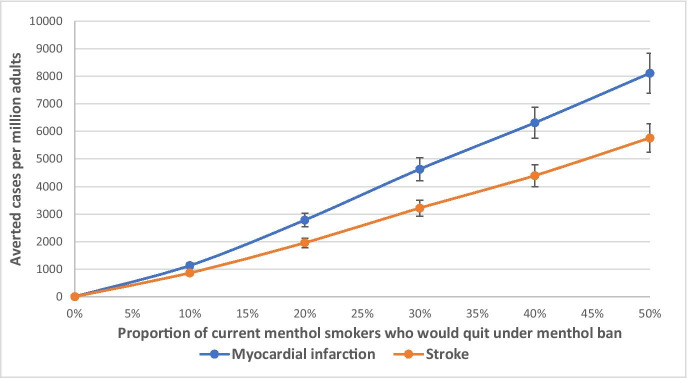
Projected cumulative averted cases of myocardial infarction and stroke in 20 years with different proportions of current menthol smokers who would quit under a menthol ban

Table 2 presents the projected number of MI and stroke events and healthcare costs among adults who smoke in NYC for the 20-year period. Under the status quo scenario (no menthol ban), the model estimated that there could be 57,232 (95% CI: 51,967–62,497) MI cases and 52,195 (47,446–56,945) stroke cases per 1 million adults who smoke in NYC by 2038. If a menthol cigarette ban policy was implemented in 2018, 2,862 MI cases and 1,983 stroke cases per 1 million adults could be averted by 2038. Our sensitivity analysis showed that, if the proportion of current menthol smokers who would quit under a menthol ban could increase to 50%, the averted cases of MI and stroke would be 8,115 and 5,762 per 1 million adults, respectively, by 2038 (Fig. 2). The model also projected that an average of $1,836 in healthcare costs per person could be saved among adults who smoke for the 20-year period due to the implementation of a menthol ban policy (Table 2). Given that the total number of adults who smoke was about 880,000 in NYC (Table 1), the total healthcare cost saving was estimated to be $1.62 billion.

**Table 2:** Projected numbers of myocardial infarctions, strokes, and healthcare costs among NYC adult smokers, 2018–2038

Policy	No. of MI, per million adults (95% CI)	Averted cases of MI, per million adults	No. of stroke, per million adults (95% CI)	Averted cases of stroke, per million adults	Healthcare costs, $ per person (95% CI)	Healthcare cost saving, $ per person
No menthol ban	57,232 (51,967, 62,497)	**––**	52,195 (47,446, 56,945)	**––**	41,479 (37,622, 45,337)	**––**
Menthol ban	54,370 (49,368, 59,372)	2862	50,212 (45,643, 54,781)	1983	39,643 (35,956, 43,330)	1836

**Fig. 3:**
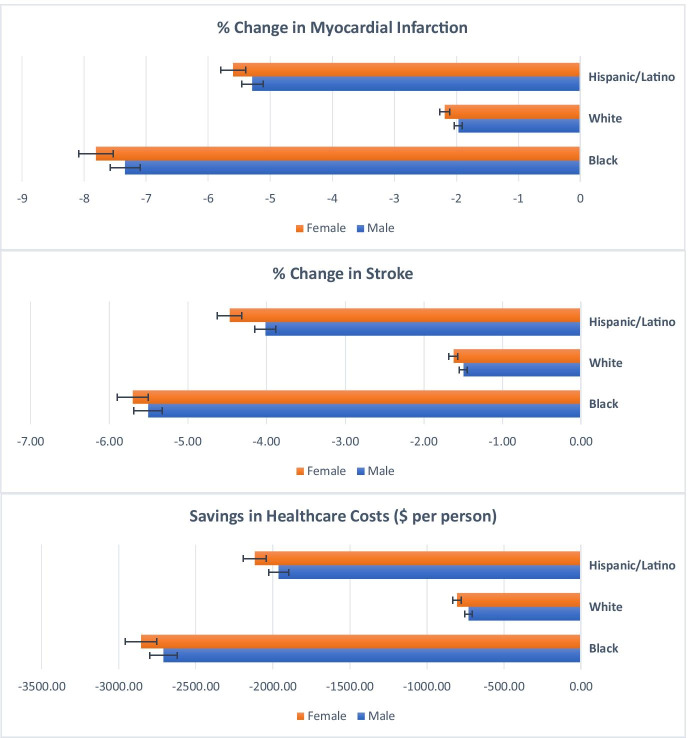
Projected reduction in incidence of cardiovascular diseases and healthcare costs by sex and race/ethnicity due to a potential menthol cigarette ban in NYC

Figure 3 reports the projected reductions in the incidence of MI and stroke and savings in healthcare cost by sex and race and ethnicity. Non-Latino Black women were estimated to have the largest reductions in the incidence of MI and stroke, while White men were to have the least reductions in both incidences. Specifically, a potential menthol cigarette ban could reduce the incidence of MI by 7.81% among non-Latino Black women, 5.60% among Latino women, and 2.19% among non-Latino White women (*p* < 0.001). The policy could reduce the incidence of MI by 7.34% among non-Latino Black men, 5.29% among Latino men, and 1.97% among non-Latino White men (*p* < 0.001). The reduction in stroke incidence follows a similar pattern; the policy was projected to have a more significant impact on non-Latino Black and Latino adults and women compared to non-Latino White adults and men.

As for healthcare costs, the menthol cigarette ban policy could save an average of $2,855 per person among non-Latino Black women who smoke, $2,116 per person among Latino women who smoke, and $805 per person among non-Latino White women who smoke over 20 years. Among men, the healthcare savings were projected to be $2,709 per person among non-Latino Black adults who smoke, $1,961 per person among Latino adults who smoke, and $730 per person among non-Latino White adults who smoke. Sensitivity analysis on discount rate showed that a higher discount rate would result in less healthcare cost saving, though the overall impact across the tested range (i.e., 0–6%) was modest (results not shown in the figure).

## Discussion

The results from our microsimulation model of the 20-year projected effects of a menthol smoking ban on CVD risk in NYC showed that the implementation of a menthol ban would result in an overall 5% reduction in MIs, a 3.8% reduction in stroke, and a cost savings of $1.62 billion over 20 years. The reduction in adverse outcomes projected by the model showed the most gains among women and particularly Black women. These results are consistent with other reports on the effect of menthol bans on health outcomes, [23] and our results project the implications of this policy on racial and ethnic disparities by 2038. To the best of our knowledge, our study is among the first to assess the impact of menthol ban policies on long-term health outcomes and disparities.

Regulatory policies to reduce smoking overall—including restrictions on advertising and smoking bans in workplaces and public spaces—have led to lower prevalence rates in smoking, but racial and ethnic disparities in menthol use persist. Menthol flavoring in cigarettes was first introduced in the late 1920s in the US, and concerted efforts by the tobacco industry have targeted women and Black communities to increase consumption among a previously low-smoking demographic. [14, 41] The result of this strategy was an increase in tobacco use among these groups in the ensuing years with a particular preference for menthol-flavored cigarettes, which is still present today. Recent studies show that menthol bans have led to a significant reduction in the sales of cigarettes and an increase in quit rates among usual and occasional menthol smokers. [22, 23] Promising results of menthol bans also show that racial and ethnic minorities report a higher rate of quitting among daily menthol smokers, [23] but barriers to uptake of evidence-based treatments remain.

Evidence-based treatments to reduce smoking have not been made accessible enough for low-income and minority groups. Racial and ethnic minority groups are particularly vulnerable to the risks of smoking, and uptake of protective strategies are less likely to be successful. [42, 43] Moreover, lack of recognition of sociocultural contexts in the implementation of social and policy interventions, including limited community engagement and collaboration with communities in developing policy interventions, may also be creating and/or exacerbating disparities. These need to be further examined and addressed to ensure that everyone in NYC has the opportunity to shape and benefit from these policy interventions.

This study has several limitations. First, like all simulation models, our model is a simplification of the real world and, thus, includes assumptions that may not be fully supported based on existing data. For example, we assumed that the menthol cigarette ban would only affect current menthol smokers rather than current non-menthol smokers or non-smokers. While a recent study has shown that the menthol cigarette ban may have negligible impact on current non-menthol smokers, [38] the policy impact on non-smokers (e.g., smoking initiation) is less clear and warrants further investigation. Also, we assumed that other health behaviors (e.g., diet, physical activity) would not change as simulated individuals age because introducing changes in other health behaviors would make it difficult for us to assess the effect of smoking on CVD. Second, we did not model the impact of smoking on non-CVD health outcomes such as lung cancer. Smoking is a risk factor for many diseases; it is impossible to model all of them. As a result, the projected health benefits and cost savings likely underestimated the other potential benefits of the menthol ban. Third, the CVD risk equations used in our microsimulation model did not include socioeconomic status (SES) such as education and income, which has been shown to be an important risk factor for CVD. [44] Thus, we were not able to model the impact of the menthol ban policy across different SES groups. More broadly, most existing CVD risk equations do not include SES. Further research should be done to incorporate SES into CVD risk equations, which will allow modeling researchers to examine the root causes of CVD health outcomes and health disparities. Additionally, our model was not able to assess the impact of a menthol ban on the number of cigarettes smoked per day or secondhand smoking due to the lack of related data and evidence. Both are important aspects related to a menthol ban and should be investigated in future epidemiological and modeling studies when new data become available. Finally, the policy environment is dynamic, and other local and federal legislations might be synergistic or antagonistic with a menthol ban. However, we were not able to capture these potential complex interactions between the menthol ban and other policies in the current study.

## Conclusion

There is national support for a menthol cigarette sales ban. [45] The health cost of smoking-related illness in the US is approximately $300 billion, and smoking results in 480,000 deaths per year. [46] In NYC, the prevalence of menthol smoking among people who currently smoke is about 50% with the highest prevalence observed among non-Latino Black individuals (84%) and the lowest prevalence among non-Latino White individuals (26%). [36] Findings from our study show a reduction in CVD morbidity and an increase in related healthcare cost savings over a 20-year period with the implementation of a menthol ban in NYC. These results may be useful for policymakers that advocate for regulatory policies that limit access to cigarettes. Further, racial and ethnic minorities have been unfairly targeted by tobacco and advertising companies, likely contributing to the smoking-related health disparities seen across these groups. Results from this study thus would support a health equity agenda aimed at reducing health disparities in racialized populations.
