# Correction to: Assessing the Health and Economic Impact of a Potential Menthol Cigarette Ban in New York City: a Modeling Study

**DOI:** 10.1007/s11524-021-00597-0

**Published:** 2021-12-10

**Authors:** Yan Li, Julia Sisti, Karen R. Flórez, Sandra S. Albrecht, Anita Viswanath, Marivel Davila, Jennifer Cantrell, Diksha Brahmbhatt, Azure B. Thompson, John Jasek, Earle C. Chambers

**Affiliations:** 1 Department of Population Health Science and Policy, Icahn School of Medicine at Mount Sinai, New York, NY 10029 USA; 2 NYC Department of Health and Mental Hygiene, New York, NY USA; 3 Environmental, Occupational, and Geospatial Sciences Department, CUNY School of Public Health and Health Policy, New York, NY USA; 4 Department of Epidemiology, Mailman School of Public Health at Columbia University, New York, NY USA; 5 Department of Epidemiology, NYU Global School of Public Health, New York, NY USA; 6 Department of Community Health Sciences, School of Public Health, SUNY Downstate Health Sciences University, Brooklyn, NY USA; 7 Department of Family and Social Medicine, Albert Einstein College of Medicine/Montefore Medical Center, 1300 Morris Park Ave, Bronx, NY USA

**Correction to: J Urban Health**

10.1007/s11524-021-00581-8

The article “Assessing the Health and Economic Impact of a Potential Menthol Cigarette Ban in New York City: a Modeling Study”, written by Yan Li, Julia Sisti, Karen R. Flórez, Sandra S. Albrecht, Anita Viswanath, Marivel Davila, Jennifer Cantrell, Diksha Brahmbhatt, Azure B. Thompson, Juhn Jasek, and Earle C. Chambers was originally published electronically on the publisher’s internet portal on 9 November 2021 without open access. With the author(s)’ decision to opt for Open Choice the copyright of the article changed on 28 November 2021 to © The Author(s) 2021 and the article is forthwith distributed under a Creative Commons Attribution 4.0 International License, which permits use, sharing, adaptation, distribution and reproduction in any medium or format, as long as you give appropriate credit to the original author(s) and the source, provide a link to the Creative Commons license, and indicate if changes were made. The images or other third party material in this article are included in the article’s Creative Commons license, unless indicated otherwise in a credit line to the material. If material is not included in the article’s Creative Commons license and your intended use is not permitted by statutory regulation or exceeds the permitted use, you will need to obtain permission directly from the copyright holder. To view a copy of this license, visit http://creativecommons.org/licenses/by/4.0/.

